# A new method for investigating bioequivalence of inhaled formulations: A pilot study on salbutamol

**DOI:** 10.3389/jpps.2023.11466

**Published:** 2023-05-03

**Authors:** Homa Rezaei, Maryam Khoubnasabjafari, Vahid Jouyban-Gharamaleki, Hamed Hamishehkar, Mohammad Reza Afshar Mogaddam, Elaheh Rahimpour, Reza Mehvar, Abolghasem Jouyban

**Affiliations:** ^1^ Pharmaceutical Analysis Research Center and Faculty of Pharmacy, Tabriz University of Medical Sciences, Tabriz, Iran; ^2^ Student Research Committee, Faculty of Pharmacy, Tabriz University of Medical Sciences, Tabriz, Iran; ^3^ Tuberculosis and Lung Diseases Research Center, Tabriz University of Medical Sciences, Tabriz, Iran; ^4^ Department of Anesthesiology and Intensive Care, Faculty of Medicine, Tabriz University of Medical Sciences, Tabriz, Iran; ^5^ Liver and Gastrointestinal Diseases Research Center, Tabriz University of Medical Sciences, Tabriz, Iran; ^6^ Kimia Idea Pardaz Azarbayjan (KIPA) Science Based Company, Tabriz University of Medical Sciences, Tabriz, Iran; ^7^ Drug Applied Research Center, Tabriz University of Medical Sciences, Tabriz, Iran; ^8^ Food and Drug Safety Research Center, Tabriz University of Medical Sciences, Tabriz, Iran; ^9^ Infectious and Tropical Diseases Research Center, Tabriz University of Medical Sciences, Tabriz, Iran; ^10^Department of Biomedical and Pharmaceutical Sciences, School of Pharmacy, Chapman University, Irvine, CA, United States; ^11^ Pharmaceutical Sciences Research Center, Shahid Beheshti University of Medical Sciences, Tehran, Iran

**Keywords:** salbutamol, bioequivalence, inhaled formulations, exhaled breath condensate, pharmacokinetics

## Abstract

**Purpose:** An efficient, cost-effective and non-invasive test is required to overcome the challenges faced in the process of bioequivalence (BE) studies of various orally inhaled drug formulations. Two different types of pressurized meter dose inhalers (MDI-1 and MDI-2) were used in this study to test the practical applicability of a previously proposed hypothesis on the BE of inhaled salbutamol formulations.

**Methods:** Salbutamol concentration profiles of the exhaled breath condensate (EBC) samples collected from volunteers receiving two inhaled formulations were compared employing BE criteria. In addition, the aerodynamic particle size distribution of the inhalers was determined by employing next generation impactor. Salbutamol concentrations in the samples were determined using liquid and gas chromatographic methods.

**Results:** The MDI-1 inhaler induced slightly higher EBC concentrations of salbutamol when compared with MDI-2. The geometric MDI-2/MDI-1 mean ratios (confidence intervals) were 0.937 (0.721–1.22) for maximum concentration and 0.841 (0.592–1.20) for area under the EBC-time profile, indicating a lack of BE between the two formulations. In agreement with the *in vivo* data, the *in vitro* data indicated that the fine particle dose (FPD) of MDI-1 was slightly higher than that for the MDI-2 formulation. However, the FPD differences between the two formulations were not statistically significant.

**Conclusion:** EBC data of the present work may be considered as a reliable source for assessment of the BE studies of orally inhaled drug formulations. However, more detailed investigations employing larger sample sizes and more formulations are required to provide more evidence for the proposed method of BE assay.

## Introduction

Inhaled formulations are highly accepted as the first-line therapy and optimal route of administration of drugs for lung diseases (1, 2). In addition to the brand inhaled formulations, there are many generic forms which require bioequivalence (BE) studies and there are some controversies in the criteria considered in the BE studies of orally inhaled formulations. The BE studies ensure that equal doses of a drug are delivered and produce equivalent pharmacological effects. For oral formulations, the blood (serum or plasma) concentration profile and the computed pharmacokinetic parameters based on this profile are the best representation. Therefore, the formulations providing comparable blood concentration profiles and pharmacokinetic parameters could be considered bioequivalent. However, this is not the case for the BE of orally inhaled formulations and establishing a generally accepted method is challenging. In the USA, *in vitro*, pharmacokinetic, pharmacodynamic and clinical endpoint studies are needed to demonstrate the BE of orally inhaled formulations. For European countries, most orally inhaled formulations are registered according to pharmacokinetic BE studies. Canadian and Japanese regulatory agencies adopt the weight of evidence approach including all aspects of cooperative testing. Chinese regulatory agency uses two strategies for registered and non-registered reference drugs in China. For the generic formulations of a reference product registered in China, they use a similar approach to European countries, and for the non-registered reference products, a new drug application is mandatory (3). These criteria are briefly reviewed in a recent work (4).

The most useful tool for comparing the BE of different inhaled formulations or different inhalation devices is the pharmacodynamics efficacy study of the drug (5, 6). The pharmacodynamics studies are performed in healthy volunteers to avoid the possible effects of past or current drug therapies, variations due to different degrees of airway inflammation and obstructive impairment (7). According to the literature (8–10), 10%–50% of the administered dose is delivered to the lung after inhalation from dry powder or metered dose inhalers (MDIs).

In a recent publication, we briefly reviewed the available methods of BE studies of inhaled formulations and compared them with the EBC concentrations of the drugs and the advantages of using EBC samples in pharmaceutical investigations (11). A new hypothesis was proposed suggesting the use of the EBC concentration profile of drugs instead of the blood concentration profiles in BE studies of the inhaled drugs (11). The main idea of this hypothesis came from the fact that all affecting parameters on drug delivery from inhaled formulations will result in drug concentrations in lung lining fluid and EBC concentration is a good representative of that concentration. In the following study, the applicability of the hypothesis was tested on the tobramycin profile in EBC. Although the results of this small-size pilot study were promising, we had two main limitations; i.e., only one tobramycin inhaled formulation was available, and we used an analytical method with low sensitivity and selectivity, which resulted in wide variations of EBC concentration profiles of six healthy volunteers (12). In another interesting work, Sadiq et al. (13) investigated the lung pharmacokinetics of several inhaled and orally administered drugs including salbutamol. They measured the levels of salbutamol in plasma, epithelial lung lining fluid (ELLF), bronchoalveolar lavage (BAL) and in some cases in the filters adsorbing the exhaled particles. The found concentrations of salbutamol in most of the filter samples were below the limit of quantification of the used analytical method. The authors concluded that the pharmacokinetic profile of drug concentration in different compartments of the human lung is feasible (13).

Aside from the sampling, using a reliable method for the determination of drug concentration is a critical point in BE investigations. The very low drug concentrations in plasma, which are near or even below the limit of detection of common analytical methods, lead to the examination of plasma or urine drug concentration using these methods. However, the plasma or urine concentrations may not be a useful tool for BE assessment of formulations containing drugs with intended local actions in the respiratory system. Despite the development of sufficient analytical methods with high accuracy, precision and capability of determination of very low concentrations of drugs, introducing straightforward methods with the aim of BE assessment of pharmaceutical products is getting more and more attention nowadays. The type of biological sample to achieve sensitive, selective, accurate and fully validated analytical techniques has been of major importance. In this aspect, exhaled breath condensate (EBC) can be regarded as an excellent sample type for the inhaled drugs and soluble components arising from the lower respiratory tract to be used for pharmacokinetic/pharmacodynamics studies (14). Moreover, non-invasiveness, cost-effectiveness and ease of operation are some clinical traits of interest associated with this type of sample collection.

Salbutamol (or albuterol) was chosen for this purpose owing to being a widely used and commercially available β₂ adrenergic receptor agonist, and previous studies on its bioavailability have been of interest (15–25). Furthermore, the total quantity of salbutamol delivered to the lung from different formulations have been investigated by plasma pharmacokinetic findings to assess the BE of generic and innovator products (22–24). Salbutamol-induced bronchodilatation has also been used to assess the relative quantity of the drug delivered to the site of action by generic and innovator formulations, however, there is no significant dose-response relationship, which causes problems in the validity of the BE assay by bronchodilatation effect (26). Stewart et al. (21) used the histamine bronchoprovocation effect of orally administered salbutamol inhalers to assess *in vivo* BE of the formulations, which provided an acceptable dose-response relationship. Rahimkhani et al. (27) investigated the aerosolization performances of a reference brand salbutamol with two Iranian generic MDIs employing *in vitro* tests. The current work is aimed to investigate the salbutamol concentration profiles in EBC of volunteers receiving two commercial salbutamol MDIs available in the market. Additionally, we measured the aerodynamic particle size distribution (APSD) parameters of the two MDIs using next-generation impactor (NGI) as an official *in vitro* test. Our goal was to use the obtained results to evaluate the BE of the MDIs and to discuss the possibility of using the proposed EBC profiles in BE studies of the inhaled formulations as a simple, low cost and efficient *in vivo* assay.

## Materials and methodology

### Chemicals

Salbutamol sulfate powder was provided by Temad Pharmaceutical Company (Tehran, Iran). 1-Flouro-2,4-dinitrobenzene and diethylethanolammonium chloride were purchased from Sigma (St. Louis, Missouri, USA). Dichloroacetic acid, pyridine, and octanoic acid were bought from Merck (Darmstadt, Germany).

### *In vitro* assessment of aerosol drug delivery

Deposition experiments were taken utilizing two commercial salbutamol MDIs (MDI-1 and MDI-2) both as sulfate salt available in the market. Both formulations are marketed to deliver 100 μg of salbutamol per puff. Immediately before each experiment, the inhaler was shaken for 15 s. The APSD of salbutamol particles of both commercial formulations was determined by employing the NGI (Coplay Scientific, United Kingdom) connected with a terminal to a critical flow controller (TPK 2000, Coplay Scientific, United Kingdom), and a vacuum pump (HCP5, Coplay Scientific, United Kingdom) to simulate the respiratory process. The hollow segments representing the oral cavity, oropharynx, larynx, trachea, carina and bronchial airways up to the fourth generation, were covered with Tween 80 (1% v/v in ethanol) as a surfactant (28), placed in an oven at 37.5°C for 20 min, then allowed to cool at room temperature. The process of actuation was repeated 10 times during each experiment (8 s pause between actuation) to facilitate the drug measurement, and experiments were performed three times at a steady flow rate of 30 L/min (29). The size distribution of each inhaler was measured from the quantity of drug recovered utilizing an appropriate volume of solvent (10 mL HPLC-grade methanol per each segment of the airway replica), and samples were maintained in conical-bottom centrifuge tubes at −80°C for further concentration analysis. Liquid chromatography-tandem mass spectrometry (LC–MS/MS) assay was developed for the determination of the mass of the drug associated with each particle size band.

### *In vivo* assessment of aerosol drug delivery

An open-label, two-way crossover study was designed and conducted at Pharmaceutical Analysis Research Center from February to November 2021. A group of nine healthy non-smoking subjects in the age group of 22-55-year-olds participated in this study. A preliminary clinical examination was done, and one subject was excluded from the study because of a family history of asthma. The project was reviewed by the Ethics Committee of Tabriz University of Medical Sciences and confirmed with the approval code of IR.TBZMED.REC.1397.695. Volunteers signed a consent form approved by the Ethics Committee of the Tabriz University of Medical Sciences, and they were well-educated about the best way to administer MDIs (30). Visits were arranged considering a washout period of 7–10 days. To avoid batch variations of MDIs (31, 32), the same two inhalers used in APSD analysis were administered to the volunteers. Immediately before each experiment, the inhaler was shaken for 15 s. In order to achieve drug concentrations well within the quantification range of the analytical technique, two actuations, 1 minute apart, were done by subjects. Immediately after inhaling the second dose, the volunteers began to exhale into the homemade EBC collector. An EBC collector (Kimia Idea Pardaz Azerbaijan (KIPA) Science Based Company, Tabriz, Iran) was employed to condensate the exhaled breath possessing tiny droplets of the airway lining fluid (ALF) at sub-zero temperatures (33). To ensure the health of the participants and prevent the risk of cross-contamination and infection, after each test, all parts of the device that came in contact with the exhaled breath were submerged in detergent-containing water for 24 h and then rinsed with double distilled water. EBC samples were collected during 0–4, 14–18, 28–32, 42–46, 56–60 and 70–74 min after inhaling the second dose and maintained in the conical-bottom microtubes at −18°C until further concentration analysis. Gas chromatography-mass spectrometry (GC-MS) was carried out for the quantification of salbutamol concentration in the EBC samples.

### Liquid chromatography-mass spectrometry of NGI samples

#### Instrumentation

Salbutamol was analyzed by a Waters Alliance HPLC (2695, Waters Milford, MA) coupled to a Waters Micromass Quattro MS/MS spectrometer (triple quadrupole tandem mass spectrometry) operating in a positive multiple reaction monitoring (MRM) mode. The MS detector was run under the following conditions: spray voltage +3 kV, source temperature 300°C, desolvation temperature 100°C, capillary voltage +2 kV, cone voltage +27 V, extractor + 3 V, electrospray mode positive, desolvation flow rate 600 L/h, and cone spray 100 L/h. The collision gas (Ar) pressure was 0.2 Pa. The transitions of 240 → 148 and 240 → 166 were used for the quantification and qualification of the analyte. The mobile phase, which consisted of a mixture of methanol and 10 mmol/L ammonium acetate (30:70, v/v), was delivered at a flow rate of 0.3 mL/min in isocratic mode.

#### Sample preparation step

The collected NGI samples were directly analyzed by LC-MS/MS system. For this purpose, 250 µL of the sample was passed through a syringe filter (0.22 μm) and injected into the column. The drug content was calculated using a calibration curve, which was developed by analyzing standards in the concentration range of 0.1–1,000 μg/mL.

### Gas chromatography-mass spectrometry of EBC samples

#### Instrumentation

Salbutamol determination was done by a GC (6890N, Agilent Technologies, Santa Clara, CA, USA)-mass spectrometer (5973, Agilent) system. The extracted/derivatized analyte was injected into the injection port adjusted at 270°C and operated in splitless/split mode. An HP-5 capillary column with a length of 30 m and film thickness of 0.5 μm) was used for the separation of the compounds. The column temperature was initially adjusted at 100°C (kept for 2 min) and increased to 270°C at a rate of 15°C/min and held for 3 min. Other conditions of MS were according to the literature (34).

#### Sample preparation step

Preparation of the samples was done according to a previously published method (34). In brief, 1 mL of diluted EBC sample was diluted with 4 mL double distilled water and mixed with 0.125 g NaCl and 20 µL pyridine to obtain a homogenous solution. Then, it was transferred into a conical bottom glass test tube, and a mixture of diethylethanolammonium chloride: dichloroacetic acid: octanoic acid deep eutectic solvent (prepared at molar ratio of 1:1:1) (55 µL), and 1-flouro-2,4-dinitrobenzene (20 µL) was added into the solution. The mixture was aspirated into a glass test tube and dispersed into the tube 5 times. After that, the cloudy solution was placed under microwave irradiation for 20 s. The mixture was centrifuged and the extracted/derivatized analytes were injected into the GC-MS system.

#### Method validation

The limit of detection (LOD), lower limit of quantification (LLOQ), linear range, precision, and extraction recovery (ER) of the method were assessed based on the US FDA (4) and ICH Registration (35) guidelines. A matrix–matched method was used for construction of the calibration curve. For this purpose, nine blank EBC samples were spiked with the analytes at the concentrations of 3, 5, 10, 25, 50, 100, 250, 500, and 1,000 ng/mL and were analyzed as described above. The coefficient of determination for the calibration curve was 0.997. The signal–to–noise ratios of 3 and 10 were used to calculate LOD and LLOQ, respectively, which were 0.5 and 1.6 ng/mL, respectively. The minimum quantifiable concentration was 1.6 ng/mL with intraday and intraday variations of 4.3 and 2.6%, respectively. Relative standard deviations of repeated analyses on the same day (*n* = 6) and different days (*n* = 6) were 2.6% and 4.3%.

### Statistical calculations

The area under the EBC-time curve (AUC) during the sampling interval was calculated using linear trapezoidal rule. Bioequivalence comparisons were made using the two one-sided t-test comparison of the log transformed ratios of C_max_ and AUC for the two MDIs with a 90% confidence interval (CI) [36]. Products were considered bioequivalent if the CIs of the log-transformed ratios of C_max_ values and ratios of AUC values of the two MDIs were within 0.8–1.25.

## Results

### *In vivo* studies

**FIGURE 1:**
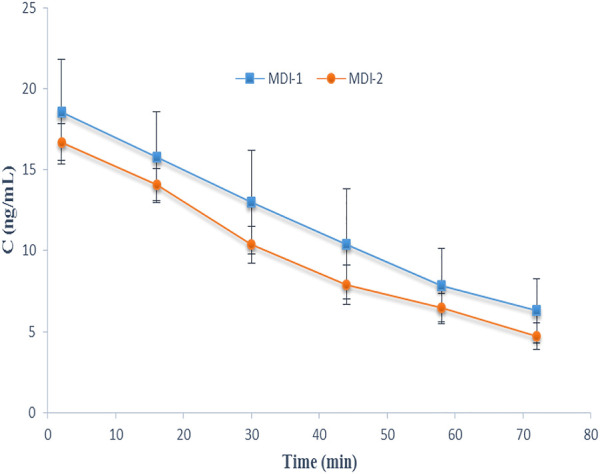
The EBC-time profiles of salbutamol after administration of the drug through MDI-1 and MDI-2 devices to 8 volunteers in a cross-over study. Data are presented as mean ± SD.

The individual characteristics of volunteers are given in Table 1. No side effects were reported by the subjects. The average EBC concentration-time courses of salbutamol for the two formulations are presented in Figure 1. Additionally, the salbutamol C_max_ and AUC values for individual volunteers after the administration of MDI-1 and MDI-2 are presented in Table 2. In all the volunteers, the C_max_ value was attained during the first exhaled breath sample collected at 0–4 min after the inhalation (Figure 1). As shown in Figure 1 and Table 2, the MDI-1 inhaler was able to produce higher concentrations and AUC of salbutamol in the lungs. The C_max_ values ranged from 11.3 to 36.3 ng/mL with a geometric mean of 17.5 ng/mL for MDI-1 (Table 2). The values for MDI-2 ranged from 11.6 to 22.3 ng/mL, with a geometric mean of 16.4 ng/mL (Table 2). The geometric mean ratio (MDI-2:MDI-1) of C_max_ was 0.937 with a CI range of 0.721–1.22 (Table 2), which fails the acceptable range of 0.8–1.25. The geometric means of AUC for MDI-1 and MDI-2 were 761 and 640 ng min/mL, with a MDI-2/MDI-1 mean ratio of 0.841 and a CI range of 0.592–1.20 (Table 2), which also fails the acceptable bioequivalence range.

**TABLE 1:** Profile of the EBC sample donors.

Volunteer	Sex	BMI	Age (year)
1	Female	21.1	24
2	Female	21.5	24
3	Male	22.6	30
4	Female	20.2	27
5	Female	19.5	23
6	Male	23.1	55
7	Female	20.7	25
8	Female	23.0	19

$BMI=\frac{Weight\;\left(kg\right)}{Height^{2}\;\left(m^{2}\right)}$ .

**TABLE 2:** The maximum concentration (C_max_) and area under the concentration-time curve (AUC) for the concentrations of salbutamol in the exhaled breath during the 2–72 min sampling period after the administration of the drug through MDI-1 and MDI-2 formulations to 8 subjects.

Subject	C_max_, ng/mL		AUC, ng.min/mL	
	MDI-1	MDI-2	MDI-1	MDI-2
1	18.3	14.2	701	483
2	11.3	19.3	391	853
3	16.5	22.3	754	952
4	13.6	11.6	670	321
5	15.2	15.6	649	686
6	36.3	17.3	1,630	778
7	16.2	16.1	753	544
8	21.3	17.2	1,030	776
GM^a^	17.5	16.4	761	640
GMR^a^	0.937		0.841	
CI^a^	0.721–1.22		0.592–1.20	

^a^ GM, geometric mean; GMR, geometric mean ratio (MDI- 2/MDI-1); CI, confidence interval.

### *In vitro* studies

Table 3 lists *in vitro* deposition fraction for major segments of the respiratory airway replica, which are the oral cavity (throat), oro-pharynx (stage 1), larynx (stage 2), trachea (stage 3), carina (stage 4), and bronchial airways up to the fourth generation. Both formulations follow a similar pattern, which is characterized by some fluctuations throughout. Apart from the oral cavity segment, in all given regions, MDI-2 takes the lead.

**TABLE 3:** *In vitro* deposition of salbutamol for MDI-1 and MDI-2 formulations in different stages of NGI.

Segment number	Segment Name	Cut size diameter at flow rate 30 L/min (µm)	MDI-1 (mg/L)	MDI-2 (mg/L)
0	Throat		172.7	129.3
1	Stage 1	11.8	5.0	7.5
2	Stage 2	6.4	3.1	4.4
3	Stage 3	4.0	6.3	8.8
4	Stage 4	2.3	16.8	21.0
5	Stage 5	1.4	11.6	15.9
6	Stage 6	0.8	4.2	3.8
7	Stage 7	0.5	1.2	6.4
8	MOC	0.4	1.2	6.6

MOC, Micro-orifice collector.

The APSD from MDI-1 and MDI-2 inhalers, which are characterized by parameters including fine particle dose (FPD), fine particle fraction (FPF), mass median aerodynamic diameter (MMDA), and geometric standard deviation (GSD) are expressed as mean ± standard deviations (*n* = 3) in Table 4. FPD values reveal the number of salbutamol particles that reached the lower respiratory system. There was no significant difference between FPD of the two MDIs studied in this work (t-test, *p* > 0.05). Considering a dose of 200 µg (two puffs, each 100 μg), FPD values of 37.4 and 33.9 µg indicate that 18.7% and 17.0% of the administered doses from MDI-1 and MDI-2 were, respectively, delivered to the lung, which are in the range of 10%–50% (8–10). A slightly more FPD value of MDI-1, in comparison with that of MDI-2, is in agreement with our *in vivo* EBC data (Table 2; Figure 1), where MDI-1 provided higher C_max_ and AUC values than MDI-2. Table 4 also lists the APSD data for other salbutamol MDIs taken from the literature (25, 27, 37–39).

**TABLE 4:** *In vitro* aerosolization performance characterization of salbutamol MDIs from the literature and this work (mean ± SD).

MDI	FPD (µg)	FPF (%)	MMAD (µm)	GSD	Reference
MDI-1	37.4 ± 13.4	16.9 ± 3.9	2.9 ± 0.3	2.4 ± 0.3	This work
MDI-2	33.9 ± 6.8	24.6 ± 1.6	2.8 ± 1.1	2.3 ± 0.9	This work
Reference (brand)	28.3 ± 5.6	30.7 ± 5.6	3.1 ± 0.4	2.7 ± 0.1	(27)
Generic I	26.2 ± 6.3	28.1 ± 4.5	3.0 ± 0.7	3.2 ± 0.2	(27)
Generic II	21.0 ± 3.7	24.1 ± 4.3	3.4 ± 0.4	2.9 ± 0.4	(27)
Ventolin®			2.4 ± 0.1	1.5 ± 0.0	(35)
Ventolin®		57.3 ± 1.3	2.4 ± 0.3	1.7 ± 0.1	(36)
Ventolin®	33.1	32.4	3.0		(25)
Test formulation	31.9	33.4	2.5		(25)
Ventolin®	26 ± 2	31 ± 2	2.4 ± 0.1	2.0 ± 0.1	(37)
ProAir®	53 ± 4	57 ± 3	2.3 ± 0.1	1.6 ± 0.1	(37)

FPD, Fine particle dose; FPF, Fine particle fraction; MMAD: Mass median aerodynamic diameter; GSD, Geometric standard deviation (replication number = 3).

## Discussion

The major purposes of the *in vitro* deposition data of the respiratory airway replica are quality control and rapid product development (40, 41). The common method, which is carried out to assess *in vitro* regional aerosol deposition from inhalers in the airway replica that includes mouth-throat (oral cavity, oropharynx and larynx) and tracheobronchial tree, is centered on applying different types of cascade impactors. The multistage liquid impinger, Anderson cascade impactor and NGI are regarded as the most widely used cascade impactors, which are recommended by both the European Pharmacopeia (EP) (chapter <2.9.18>) and the United States Pharmacopeia (USP) (chapter <601>) (42). Median aerodynamic parameter (MMAD), fine particle fraction (FPF), fine particle dose (FPD) and geometric standard deviation (GSD) are key parameters employed for the characterization of a pharmaceutical inhaler as a regulatory requirement. FPD is the fraction of drug particles’ mass that has an aerodynamic size of less than 5 μm. Such particles are small enough to enter the lung and theoretically represent a deposition pattern in the deep lung after inhalation; whereas, the term FPF refers to the situation in which this quantity is expressed by the percentage of inhalation (43). FPF may be introduced as a percentage of either metered (ex-valve) doses or delivered (ex-device) doses (44). The MMAD is defined as the diameter at which 50% of the particles by mass are larger and 50% are smaller. GSD is a measure of the sharpness of the cut of an impactor, equal to the square root of the ratio of the particle diameter, corresponding to 84.1% collection efficiency to the particle diameter corresponding to 15.9% collection efficiency.

Wide variations of drug concentrations in EBC were observed in many published works. The mean values and standard deviations for a concentration-time profile of tobramycin in EBC were reported in earlier work (12). The variations of tobramycin in EBC were also reported in another work (45). Fluctuations in the number of aerosol particles trapped in the EBC sampling device could be considered one of the reasons for obtaining varied tobramycin concentrations in EBC (46). A similar pattern has been reported for tramadol and its main metabolite in EBC (47). In addition to EBC, such wide variations have been also reported for tobramycin in BAL; 0–0.30 μg/mL (48), <0.1–9.21 μg/mL (2.0 ± 2.66 μg/mL) (49) and 3.4 ± 1.23 μg/mL (50). Larger variations (90 ± 54 μg/mL) have been observed in BAL collected from young children with cystic fibrosis after inhalation of 180 and/or 300 mg tobramycin (51). Poor reproducibility and high variability were also observed for biomarker quantifications in EBC (52).

In the process of collecting EBC samples, the ALF arises from the lower respiratory system and passes through the pharynx and mouth. As a result, the risk of exhaled breath contamination is relatively high (14). Moreover, high variability and poor reproducibility are analytically important features that limit the application of EBC for biomarker quantification (52). However, some investigations reported some techniques to decrease the variability (53–56).

Gravitational sedimentation, inertial impaction and Brownian diffusion augmented by the overall outcome of electrostatic attraction, turbulent flow and direct interception are involved in aerosol particle diffusion in the human respiratory tract (57). However, the role of bronchial circulation in a redistribution of the inhaled drug (1), age, duration of illness, gender, the type of inhaler employed (58, 59), breathing pattern (60), particle properties (61) and drug release pattern that is affected by different crystalline forms of the inhaled medication should be considered in assessing an inhaled drug’s effectiveness.

Difficulties in the process of measuring clinical endpoints and concluding for discrimination between the efficacy of different inhaled products (brand and generic formulations) lead us to quantify the deposited amount of drug in the lungs by designing efficient, cost-effective and non-invasive *in vivo* studies (40, 62). There are several confounding parameters that should be considered including; 1) formulation factors (drugs and excipients physico-chemical properties, manufacturing process and amount of excipients), 2) device factors (shape, size, external design attributes, metering method, energy source and airflow resistance, 3) patient factors (age, gender, training, mucociliary clearance efficiency and disease severity, 4) formulation-patient factors (pulmonary retention time, drug’s solubility and dissolution), 5) formulation-device factors (single actuation content, aerodynamic particle size distribution and aerosolization efficiency) and 6) patient-device factors (user interface, inhalation effort and regional deposition) (63).

Although for effective drug delivery, either systemic or local therapies, inhaled medications should be targeted to specific areas of the thoracic region, a significant fraction of the dose is deposited in the extrathoracic region generally taken to include the upper part of the trachea, larynx, pharynx, buccal cavity and nasal passages (64, 65) by inertial impaction mechanism and is partly responsible for unwanted side-effects (32). Oropharyngeal filtering is a major determinant for both the quantity of lung deposition and its variability; the higher the lung deposition, the smaller the variability in lung dose (66).

Despite all difficulties to establish a correlation between *in vivo* and *in vitro* data for oral inhalers, BE studies are considered a topic of increasing importance for patients, clinicians, drug developers and regulatory agencies (11, 32, 67). *In vitro-in vivo* correlations allow us to be more confident about predictions of the *in vivo* behavior of future inhaled formulations using *in vitro* data. In addition, these studies are welcomed by pharmaceutical companies because conducting *in vivo* data are often expensive and time-consuming because they may require a large number of patients (68). Various aspects of *in vitro—in vivo* correlations (IVIVCs) were comprehensively reviewed by Chow et al. (69). Establishing clear IVIVC is not straightforward. *In vitro* assessment of regional deposition of pharmaceutical inhalers is centered around the determination of APSD and delivered dose (42), which enables us to predict likely regional lung deposition downstream by using obtained data as an input to a numerical model (70). APSD measurement as a regulatory requirement is generally done utilizing impactors that fractionate the mass of the drug into a series of particle size bands according to their aerodynamic compartment and collect them in a series of impaction plates (32). Next-generation impactors are the most commonly used systems to study APSD, but for low density and/or high dose formulations, the Anderson cascade impactor or even multistage liquid impinger is more appropriate (71).

In brief, for this work, The MDI-1 inhaler induced slightly higher EBC concentrations of salbutamol when compared with MDI-2. The geometric MD-2/MD-1 mean ratios (confidence intervals) were 0.937 (0.721–1.22) for maximum concentration and 0.841 (0.592–1.20) for area under the EBC-time profile, indicating a lack of BE between the two formulations. In agreement with the *in vivo* data, the *in vitro* data indicated that the fine particle dose (FPD) of MDI-1 was slightly higher than that for the MDI-2 formulation. However, the FPD differences between the two formulations were not statistically significant.

## Conclusion

In conclusion, EBC concentration profiles more closely reflect human breathing patterns in comparison with the constant flow *in vitro* investigations (32). Our proposed method based on the EBC matrix was employed for the BE study of two commercially available salbutamol inhalers, and salbutamol concentrations were successfully quantified by commonly used analytical techniques. Our developed method seems to be promising for future *in vivo* studies specifically for drugs with a local effect in the lung. More detailed investigations employing larger sample sizes and more formulations are required to provide more evidence for our proposed method of BE assay.

## Data Availability

The original contributions presented in the study are included in the article/supplementary material, further inquiries can be directed to the corresponding author.
